# Effect of the N-terminal extension in myosin essential light chain A1 on the mechanism of actomyosin ATP hydrolysis

**DOI:** 10.1016/j.jbc.2023.105521

**Published:** 2023-12-01

**Authors:** David H. Heeley, Betty Belknap, Jennifer L. Atherton, Stephanie C. Hasan, Howard D. White

**Affiliations:** 1 Department of Biochemistry, Memorial University, St John’s, Newfoundland, Canada; 2 Department of Physiological Sciences, Eastern Virginia Medical School, Norfolk, Virginia, USA

**Keywords:** myosin essential light chains A1 and A2, ATPase kinetics, thin filament regulation

## Abstract

Myosin essential light chains A1 and A2 are identical isoforms except for an extension of ∼40 amino acids at the N terminus of A1 that binds F-actin. The extension has no bearing on the burst hydrolysis rate (M-ATP → M-ADP-Pi) as determined by chemical quench flow (100 μM isoenzyme). Whereas actomyosin-S1A2 steady state MgATPase (low ionic strength, 20 °C) is hyperbolically dependent on concentration: *V*_*max*_ 7.6 s^−1^, *K*_*app*_ 6.4 μM (F-actin) and *V*_*max*_ 10.1 s^−1^, *K*_*app*_ 5.5 μM (native thin filaments, pCa 4), the relationship for myosin-S1A1 is bimodal; an initial rise at low concentration followed by a decline to one-third the *V*_*max*_ of S1A2, indicative of more than one rate-limiting step and A1-enforced flux through the slower actomyosin-limited hydrolysis pathway. In double-mixing stopped-flow with an indicator, Ca(II)-mediated activation of Pi dissociation (regulatedAM-ADP-Pi → regulatedAM-ADP + Pi) is attenuated by A1 attachment to thin filaments (pCa 4). The maximum accelerated rates of Pi dissociation are: 81 s^−1^ (S1A1, *K*_*app*_ 8.9 μM) *versus* 129 s^−1^ (S1A2, *K*_*app*_ 58 μM). To investigate apomyosin-S1-mediated activation, thin filaments (EGTA) are premixed with a given isomyosin-S1 and double-mixing is repeated with myosin-S1A1 in the first mix. Similar maximum rates of Pi dissociation are observed, 44.5 s^−1^ (S1A1) and 47.1 s^−1^ (S1A2), which are lower than for Ca(II) activation. Overall, these results biochemically demonstrate how the longer light chain A1 can contribute to slower contraction and higher force and the shorter version A2 to faster contraction and lower force, consistent with their distribution in different types of striated muscle.

A molecule of striated muscle myosin is a hexamer of two heavy chains and four light chains. Each head contains one essential (alkali) light chain (ELC) and one regulatory light chain. The light chains clamp onto the heavy chain’s lever arm, with the ELC being more proximal to the active site (1, 2).

Like other sarcomeric proteins, the ELC is a family of isoforms, a tool box out of which a given member is selected to create a different functional phenotype. Two ELCs, historically termed A1 (or LC1f) and A2 (or LC3), exist in fast skeletal muscle fibers (3, 4). Three isoform combinations, A1A1, A2A2, and A1A2, have been detected (5, 6, 7). The A2 variant is absent in heart and slow skeletal muscle. These muscle types synthesize ELCs that are homologous to A1 (myosin light chain family reviewed in (8)).

Protein sequencing (9) showed that ELCA1 (source, rabbit skeletal muscle) possesses a sequence of just over 40 amino acids at the N-terminal end that is missing in ELCA2. The extension (N-ELCA1) is enriched in proline, alanine, and lysine and accounts for electrophoretic (3) and chromatographic (10) separations as well as actin binding as demonstrated by cosedimentation (11), cross-linking (12, 13, 14) and NMR (15, 16, 17). N-ELCA1 starts with a rare posttranslationally modified amino acid, trimethylalanine (18). The trimethylalanine and adjacent lysines are important for interaction with actin (19, 20) at a site that is near to actin’s C terminus (12, 17, 21, 22). A cluster of four lysines in the first eight amino acids of N-ELCA1 is followed by two motifs, a polyalanine sequence that lengthens with increasing animal body mass (23) and several Ala-Pro repeats. Downstream of the extension, which is protracted in conformation (24), is a block of ∼140 amino acids that is identical to both ELCs (9) and is required for myosin heavy chain binding. The two primary structures are completed by a small, nonidentical peptide that is sandwiched between the above two sections in A1 and which is N-terminal in A2 (9). Subsequently, the ELCs were shown to be products of a single gene (25, 26) which has been given the designation MYL1 (reviewed in (8)). Designations for other striated muscle ELC genes are the following: MYL3 (slow skeletal 1b and ventricle), MYL4 (atrium), and MYL6B (slow skeletal 1a) (8).

ELC function has been investigated in various ways. Removal of the entire light chain, something that was initially difficult to do, disrupts *in vitro* actin filament motility (27), and force production (28). Chromatographic separation (10) paved the way for a comparison of isoenzymes. Myosin-S1A2 produces the greater effect in terms of actin activation of MgATPase (first reported in (10)) and actin filament sliding velocity (29). On the other hand, the A1-containing isoenzyme is more restrictive of actin filament motion (30) and generates a larger power stroke (22). Truncation of part or all of N-ELCA1, increases actin activation (31), alters the relative positions of thick and thin filaments (32), and reduces the myosin step-size as determined by Q-dot assay (33). Another approach has involved disruption of the ELC-actin interaction with synthetic peptides and the expression of mini genes. Force production and shortening velocity were enhanced by the first 20 residues of ventricle ELC (34). Myofibrillar MgATPase was enhanced by another peptide containing residues 5 to 14 (35). A positive inotropic effect was observed following minigene expression of the first 15 amino acids of cardiac ELCs (36). In the same study, each cardiac (atrial and ventricle) ELC peptide is bound to F-actin in the micromolar range in a sedimentation assay, with the ventricle peptide displaying the higher affinity (36). Collectively, these observations form a picture in which the light chain extension serves as a spacing and guiding arm that presets the myosin head on the actin filament as well as a regulator. Of the cardiomyopathy mutations identified to date, most occur downstream of residue 50 (reviewed in (37)). In one of these cases, Glu56Gly (source, human), hypercontractility has been explained by an increase in the fraction of strongly bound, force generating heads (38).

In the present work, various individual steps in the ATP hydrolysis pathway are biochemically targeted in solution assays featuring myosins differing only in their ELC. Dissection of the pathway in this manner goes to the crux of the functional relevance of ELC isomorphism. Specifically, how the N-terminal extension influences myosin activity and in turn, muscle performance. Experiments include direct measurement of the rate of ATP bond scission (M-ATP → M-ADP-Pi) by chemical quench-flow at a sufficiently high myosin-S1 concentration where substrate binding is nonrate limiting. Under these conditions, myosin-S1A1 and -S1A2 are equivalent. Conversely, differences are apparent in F-actin activation of steady state MgATP hydrolysis (low ionic strength, 20 °C) which have ramifications for how ELC isotype may impact contractility. Whereas myosin-S1A2 MgATPase is hyperbolically dependent on concentration, the relationship for myosin-S1A1 MgATPase is characterized by an initial rise at low concentrations of F-actin (unregulated and regulated) followed, upon continued titration, by a decline to a stable level which is below that of the A2-containing isomyosin. The observations are explained by a transition from a myosin-limited hydrolysis mechanism to an actomyosin-limited hydrolysis mechanism, in which the N-terminal extension in ELCA1 increases the probability of the hydrolytic step occurring while the A1 head is attached to actin (Fig. 1, below).

**Figure 1:**
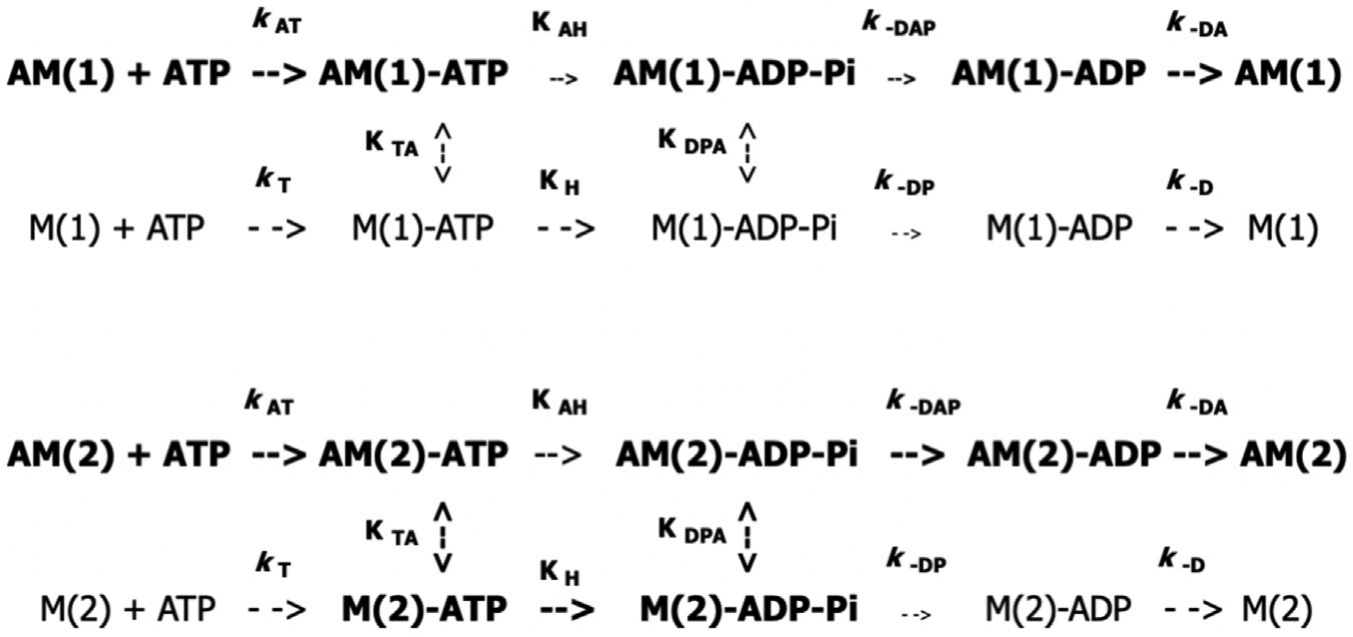
**Comparison of ATP hydrolysis pathways for isomyosins A1 and A2.** A minimal scheme is presented in which the classical Lymn-Taylor mechanism (56) is assumed to apply to both ELCA1-containing (M1) and ELCA2-containing (M2) myosins but the fraction of hydrolysis occurring along each pathway varies depending on the ELC isoform. Under the ionic strength used in the current experiments and the exceedingly high actin concentration in muscle, the N-terminal light chain extension increases the affinity of myosin intermediates for actin and enforces comparatively more flux through an actomyosin-limited hydrolysis mechanism (indicated in *bold type*) that is characterized by slower cleavage (K _*AH*_, *small arrow*) and slower Pi dissociation (*k* _-*DAP*_, *small arrow*). M(2) will promote greater flux through a myosin-limited hydrolysis mechanism that is characterized by faster cleavage (K _*H*_) and faster Pi dissociation (*k*_-DAP_). ELC, essential light chain.

Double-mixing stopped-flow fluorescence is employed to measure the rate of Pi dissociation from a pre-power stroke complex (AM-ADP-Pi → AM-ADP + Pi) composed of a given isomyosin. This particular step in the actomyosin cycle coincides with a structural transition between myosin intermediates. It is also a regulatory focal point. Previous studies carried out with skeletal myosin-S1A1 (39, 40) and cardiac myosin-S1 (41), but not skeletal myosin-S1A2, have demonstrated that the rate of Pi dissociation is accelerated by the binding of Ca(II) and apomyosin-S1 (rigor) to thin filaments and that the cardiac regulatory system is highly responsive to Ca(II) (reviewed in (42)). Using the same fast-mixing methodology, the maximum accelerated rate of Pi dissociation is 50% higher for S1A2 *versus* S1A1 (Fig. 1) but requires a seven-fold higher concentration of cardiac thin filaments (pCa 4) to attain saturation. Further, for both types of myosin-S1, greater maximal acceleration of the Pi dissociation rate is induced by bound Ca(II) alone (*i.e.* no activation by apomyosin-S1) than bound apomyosin-S1 alone (*i.e.* plus EGTA, no activation by Ca(II)).

## Results

### Chromatographic separation of skeletal myosin isoenzymes

**Figure 2:**
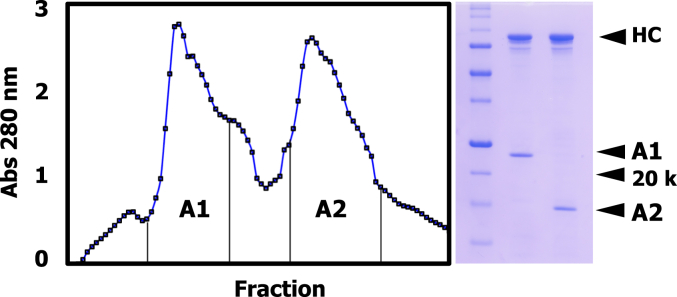
**Diethylaminoethyl-cellulose chromatography of rabbit skeletal myosin-S1.** Isoenzyme-containing fractions are pooled as indicated and processed as in Experimental procedures. Coomassie R-250-stained Laemmli gel: *left*-hand lane, markers (Bio-Rad, catalog no. 1610363), 250, 150, 100, 75, 50, 37, 25, 20, 15, and 10 kDa; central lane, S1A1 and *right*-hand lane, S1A2. The positions of myosin-S1 heavy chain (HC), ELCs and 20 kDa marker are indicated by chevrons in *right*-hand margin. Differential ELC staining intensity is attributed to differences in amino acid composition, notably lysine. ELC, essential light chain

Following limited digestion of rabbit skeletal muscle myosin with chymotrypsin, the liberated, soluble head fragment is fractionated into two pools by passage over diethylaminoethyl-cellulose (Fig. 2). Consistent with the difference in lysine content of the two ELCs (9), the earlier eluting peak contains myosin-S1A1 whereas the later peak contains myosin-S1A2. In accordance with their different lengths, the ELCs run above (A1) and below (A2) the 20 kDa marker during SDS PAGE (Fig. 2). Following dialysis, the two myosin-S1 isoenzymes are deployed in various kinetic experiments.

### Effect of essential light chain isotype on rate of ATP cleavage by myosin-S1

**Figure 3:**
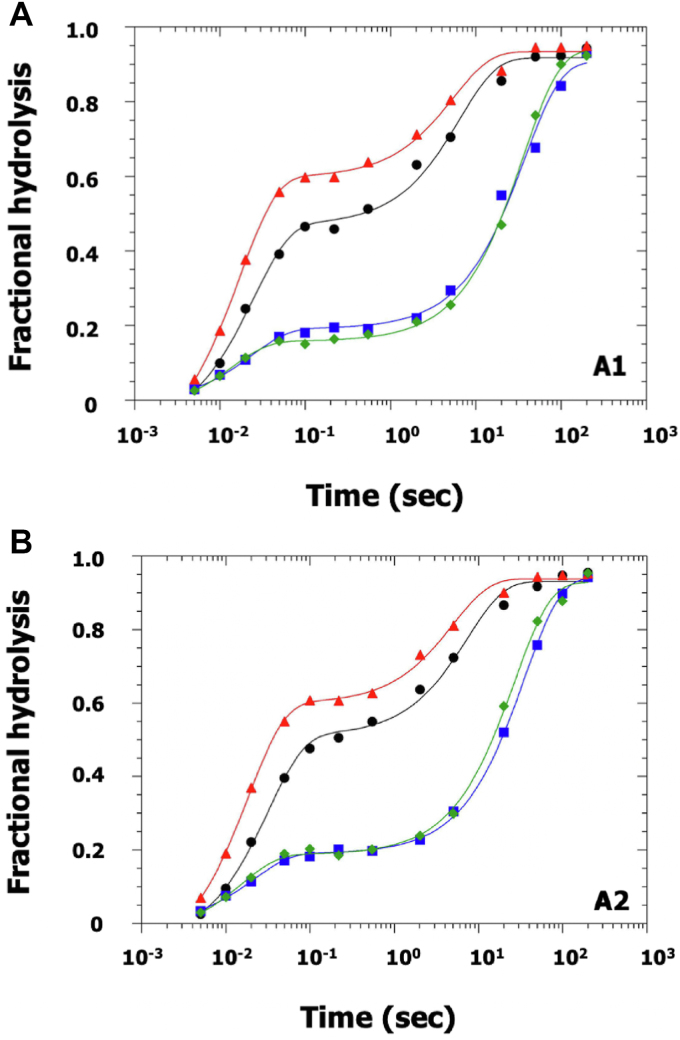
**Chemical quench-flow analysis of ATP hydrolysis by skeletal myosin-S1 containing either ELCA1 or ELCA2.** A given myosin-S1 is mixed with an equal volume of MgATP containing γ-^32^P-ATP in different molar ratios and allowed to incubate for 0.005 to 200 s. Samples are nonblind and nonrandomized. Fractional hydrolysis is obtained from the ratio of the charcoal-treated and total counts. Symbols, *red triangles* (100 μM S1 + 25 μM ATP), *black circles* (50 μM S1 + 12.5 μM ATP), *blue squares* (50 μM S1 + 200 μM ATP) and *green diamonds* (100 μM S1 + 400 μM ATP). Stated concentrations are final, specifically half of the starting amount. Note: the amounts of protein used did not permit technical replicates. Curves are fit by equations with two exponential terms to determine the pre-steady state and steady state rates of hydrolysis. Fitting parameters for myosin-S1A1 in plot (*A*): *red*, f(t) = 1.0-(0.62e^-58.4^^∗^^t^ + 0.33e^-0.18∗t^). *black*, f(t) = 1.0-(0.54e^-40.7∗t^ + 0.45e^-0.16∗t^). *blue*, f(t) = 1.0-(0.19e^-43∗t^ + 0.78e^-0.027∗t^). *green*, f(t) = 1.0-(0.19e^-43∗t^ + 0.71e^-0.027∗t^). Fitting parameters for myosin-S1A2 in plot (*B*): *red*, f(t) = 1.0-(0.69e^-54.1∗t^ + 0.33e^-0.20∗t^). *black*, f(t) = 1.0-(0.56e^-31.5∗t^ + 0.42e^-0.14∗t^). *blue*, f(t) = 1.0-(0.19e^-65.9∗t^ + 0.74e^-0.038∗t^). *green*, f(t) = 1.0-(0.19e^-47∗t^ + 0.75e^-0.029∗t^). ELC, essential light chain.

The hydrolysis of ATP by myosin-S1 in the absence of actin is analyzed by chemical quench-flow (T, 20 °C). The method affords a direct measure of the rate of scission or “burst” (M-ATP → M-ADP-Pi) during the first turnover. To ensure a meaningful measurement, the concentration of myosin-S1 must be sufficiently high so not to be limited by substrate binding (M + ATP → M-ATP). Accordingly, mixtures contain a final concentration of either 100 or 50 μM enzyme in four-fold molar excess over substrate (red and black symbols, Fig. 3, *A* and *B*) as well as a four-fold molar excess of substrate over enzyme (blue and green symbols, Fig. 3, *A* and *B*). Thus, four combinations per isoenzyme.

Mixtures are acid quenched at times from 0.005 to 200 s with a total of seven incubations in the millisecond range. Two distinct phases are evident in plots of fraction of hydrolyzed substrate *versus* time (Fig. 3, *A* and *B*). The fast phase, which is completed prior to 1 s, arises from the “burst” hydrolysis of ATP (M-ATP → M-ADP-Pi). The slow phase arises from steady state hydrolysis and is limited by Pi release. In both instances, the fraction hydrolyzed approaches completion at the longest incubation times (Fig. 3, *A* and *B*).

Equations for biexponential fits of the profiles are provided in the legend for Figure 3, *A* and *B*. There is little difference between the two myosin-S1s. At the highest concentration of enzyme, 100 μM, and 25 μM ATP (red symbols) and 400 μM ATP (green symbols), the observed burst hydrolysis rates fall within a similar range, 43 to 58 s^−1^ (S1A1) and 47 to 54 s^−1^ (S1A2) and are clearly slower than the rate of ATP binding, estimated to be >400 s^−1^. It should be noted that this information was not provided in the original study of Taylor and Weeds (43), due to a low enzyme concentration and time resolution. In agreement with the original study (43), we find that myosin-S1A1 and S1A2 hydrolyze MgATP at essentially the same rate. As an example, for the condition of 100 μM S1 + 400 μM ATP the corresponding values are 0.027 s^−1^ (S1A1) and 0.029 s^−1^ (S1A2). Further, when there is excess enzyme and all of the substrate can be expected to be bound, for example, 100 μM myosin-S1 + 25 μM ATP, the phase amplitudes correspond to the proportions of bound products and bound unhydrolyzed substrate and the ratio is a measure of the equilibrium constant, *K*_*eq*_ 1.9 (S1A1) and 2.1 (S1A2). Thus, under conditions where substrate binding is not rate limiting, the ELC isoform has minimal, if any, effect on the kinetics of skeletal myosin-S1 ATP hydrolysis in the absence of actin.

### Effect of essential light chain isotype on the actomyosin-S1 steady state MgATP hydrolysis rate

**Figure 4:**
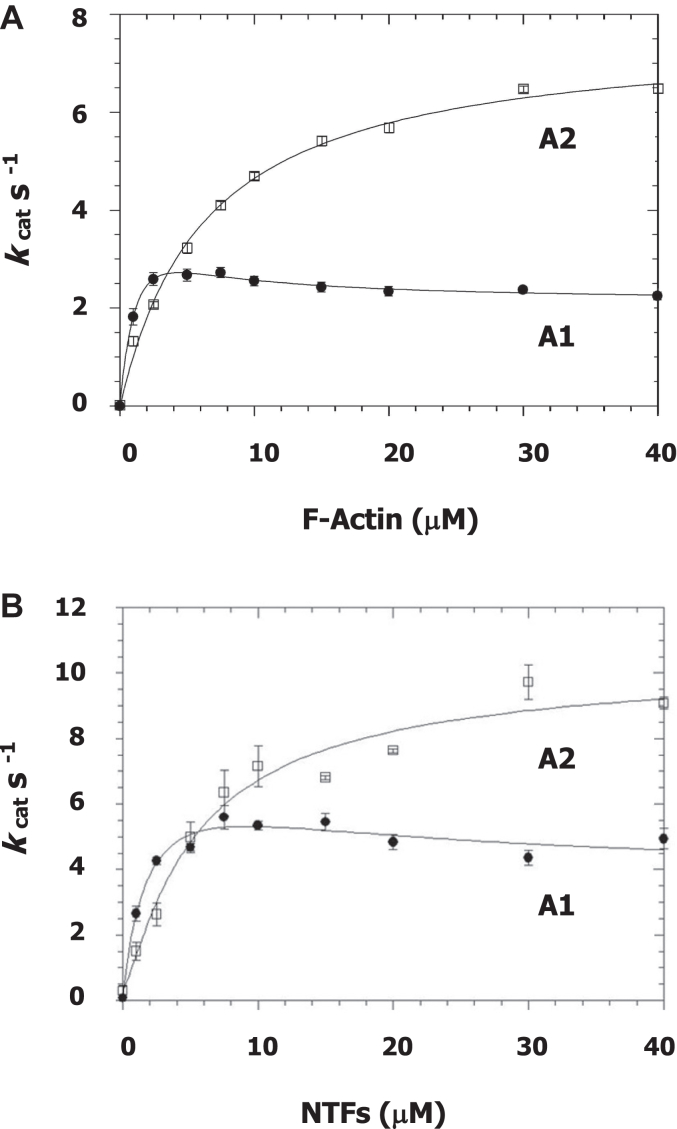
**F-actin and native thin filament (pCa 4) dependence of the steady state MgATPase rate of rabbit skeletal myosin-S1 containing either ELCA1 or ELCA2**. Shown are averages of technical replicate titrations performed on the same day. Samples, nonblind and nonrandomized. *A*, unregulated actin, n = 7. *B*, native thin filaments (NTFs) at high [Ca(II)], n = 3. *t* test comparisons of rates (A1 *versus* A2) yield *p* values < 0.01 (zero–3 μM F-actin) to < 0.001 (10–40 μM F-actin). Experimental conditions, 5 mM Mops, 2 mM Mg(II), 1 mM MgATP, pH 7.00, 20 °C. Initial rates are obtained from multiple time points. In the lower F-actin concentration range, 0.12 μM myosin-S1 is used and data are collected between zero and 15 min. In the higher range, 0.061 μM myosin-S1 is used and data are collected between zero and 7.5 min. The plotted relationship for myosin-S1A1 is obtained by fitting to the appropriate steady state kinetic equation using a nonlinear least squares simplex operation (90). ELC, essential light chain.

Experiments are carried out in a low ionic strength buffer without additional salt (5 mM Mops, 2 mM Mg(II), pH 7.00, T 20 °C. The steady state rate of MgATP hydrolysis by the A1-containing isoenzyme (closed symbols) displays a nonhyperbolic dependence on the concentration of unregulated F-actin (Fig. 4*A*). At low concentrations, there is an initial rise bearing a slope of 1.5 μM per sec followed by a decrease to a stable level with increasing actin concentration. Specifically, the fit curve reaches a value of 2.75 s^−1^ at 4 μM F-actin. The projected maximum is judged to be >4 s^−1^ at 6 μM but is not accurately determined. As the F-actin concentration is raised the rate falls, reaching a value of 2.3 s^−1^ at 40 μM. Note: these values, obtained at a temperature of 20 °C, are higher than those reported previously at 10 °C (44) where a bimodal relationship for myosin-S1A1 was also reported (44). Over the same concentration range, activation of the A2-containing isoenzyme (open symbols) conforms to a hyperbola having a maximum rate of 7.6 s^−1^ (Fig. 4*A*). The maximum is attained at a higher F-actin concentration (*K*_*app*_, 6.4 μM) than what is observed with S1A1 (*K*_*app*_ < 2 μM). When measurements are carried out using native thin filaments (NTFs) containing bound Ca(II), bimodal and hyperbolic concentration-dependences are again observed (Fig. 4*B*). The fit curve for myosin-S1A1 crests at around 5 s^−1^ at 6 μM NTFs and then declines to an extrapolated *V*_*max*_ of 4 s^−1^. By comparison, the curve for myosin-S1A2 extrapolates to a *V*_*max*_ of 10.1 s^−1^ (K_app_, 5.5 μM). Thus, maximal activation of the A2 isoenzyme by F-actin and by NTFs (pCa4) is roughly three times that of the A1. Further, in both experiments (Fig. 4, *A* and *B*), the more rapid ATPase at low actin monomer concentration and the lower maximum ATPase rate at high actin monomer concentration observed with S1 containing the A1 light chain can be attributed to the comparatively tighter binding of the A1 head to actin/thin filaments which, in turn, favors a slower “attached” hydrolysis route (upper pathway in Fig. 1).

### Effect of essential light chain isotype on the Ca(II)-accelerated rate of Pi dissociation from a native thin filament-myosin-S1-ADP-Pi complex

**Figure 5:**
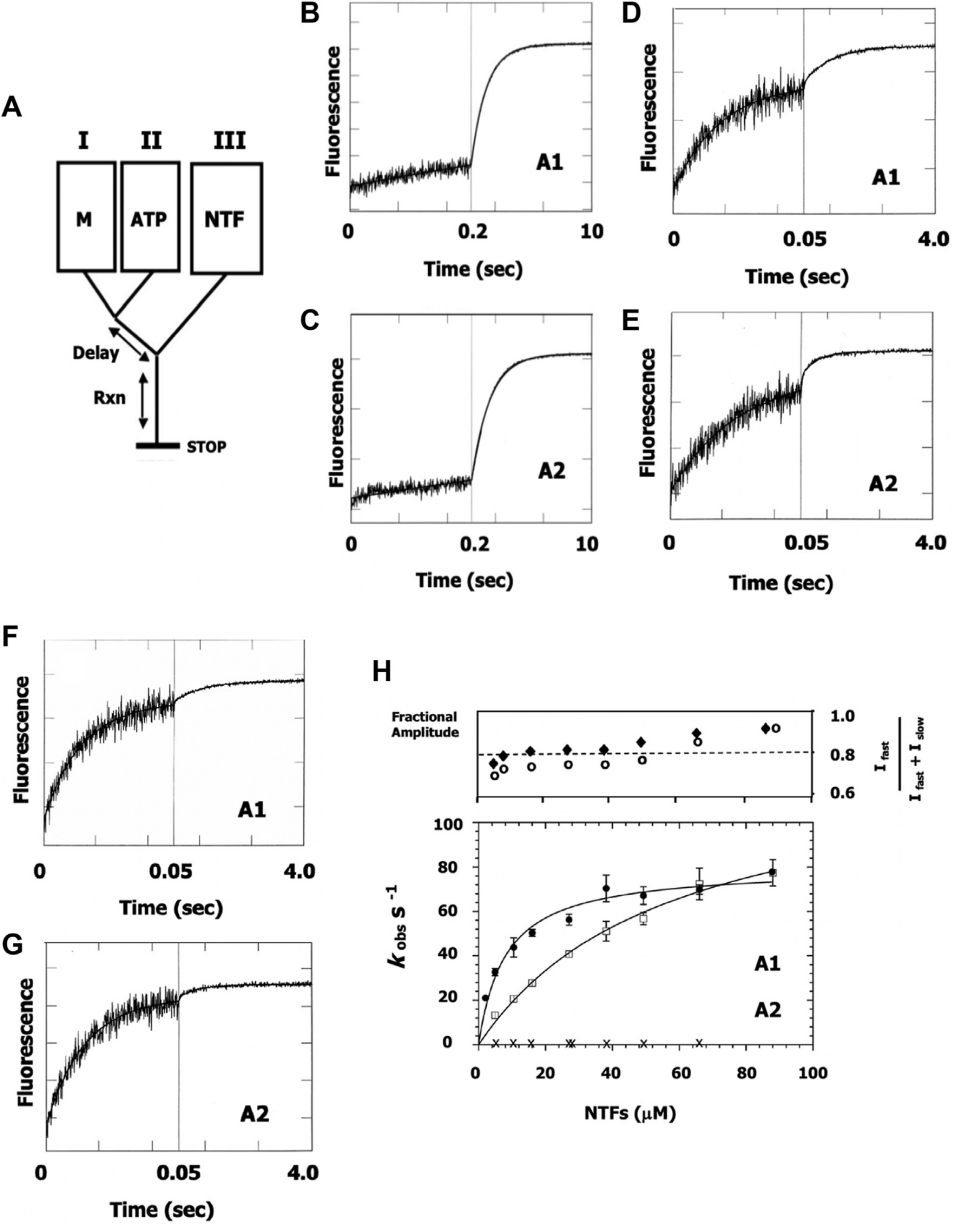
**Inorganic phosphate dissociation from a thin filament-myosin-S1-ADP-Pi complex at low and high [Ca(II)] and in the absence of apomyosin-S1.***A*, schematic of double-mixing stopped-flow instrument configuration. Syringe “I”, myosin-S1A1 or S1A2 (M); syringe “II”, ATP and syringe “III”, native cardiac thin filaments (NTFs). Delay between mixes, 1 s. Buffer, 5 mM Mops, 2 mM Mg acetate, pH 7.00, 20 °C. All solutions contained Pi binding protein, 0.01 units/ml purine nucleoside phosphorylase and 0.1 mM 7-methylguanosine. *B*–*G*, time traces. Pi release is monitored over two different time windows each of 250 data points, in accordance with the process that is being monitored. In each instance, the presented time trace is an average of recordings stemming from three or more consecutive shots (*i.e.* passes through the stopped-flow instrument). Concentrations are adjusted for mixing dilutions as specified in Experimental procedures. Comparison of isomyosins (nonblind) is performed on the same day. *B* and *C*, time traces for turned-off NTFs (pCa 8, zero apomyosin-S1). Final concentrations in flow cell, 0.44 μM myosin-S1, 0.44 μM ATP, 27 μM NTFs, 1.1 mM EGTA and 5 μM phosphate binding protein. The presented traces are best fits to the following single exponential equations: (*B*) I(t) = 1.0 e^-0.8t^ + C and (*C*) I(t) = 1.0 e^-0.66t^ + C. *D*–*G*, time traces for Ca(II)-activated NTFs (pCa 4, zero apomyosin-S1). Final concentrations in flow cell, 1.1 μM myosin-S1, 0.66 μM ATP, 27 μM NTFs (*D* and *E*) or 65 μM NTFs (*F* and *G*) 0.3 mM CaCl_2_ and 10 μM phosphate binding protein. The presented traces are best fits to the following double exponential equations: *D*, I(t) = 0.73 e^-55t^ + 0.27 e^-1.2t^ + C. *E*, I(t) = 0.81 e^-40.5t^ + 0.19 e^-2.1t^ + C. *F*, I(t) = 0.84 e^-68.8t^ + 0.16 e^-1.2t^ + C. *G*, I(t) = 0.89 e^-69t^ + 0.11 e^-3.4t^ + C. *H*, concentration dependence. Three titrations are performed using two different batches of isomyosin-S1s and NTFs. Titration is performed systematically in descending order of concentration. Points are averages of at least six shots. *Upper panel*, fractional amplitude of fast component of the fluorescence increase, I _fast_/I _fast_ + I _slow_. Fractional amplitudes are averaged and presented separately to avoid cluttering of data. *Lower panel*, concentration dependence of the fast increase in fluorescence (*D*–*G*). Hyperbolic fits yield the following parameters: S1A1 (*closed symbols*), *k*_fast_ = 81.4 s^−1^ and *K*_*app*_ = 8.9 μM; S1A2 (*open symbols*), *k*_fast_ = 129.3 s^−1^ and *K*_*app*_ = 57.6 μM. *t* test comparisons of the rates of Pi dissociation (A1 *versus* A2): *p* < 0.01 between 0 to 50 μM NTFs. At higher concentrations, *p* > 0.5 indicating convergence. Note: the slow increase in fluorescence (*D*–*G*) is concentration independent. Interpretation of the slow phase is complicated by there being more than one possible route of breakdown of the M-ATP pool including attached hydrolysis (K _AH_, Fig. 1). Crosses “x”, turned-off native thin filaments (pCa 8, zero apomyosin-S1) with myosin-S1A2 only, except for the points at 27 μM which are taken from panels B (S1A1) and C (S1A2). NTF, native thin filaments.

Experiments in the next two Results sections feature thin filaments in different liganded states. An instrument schematic is provided in Figure 5*A*. First, myosin-S1A1 or S1A2 (in syringe “I”) is combined with MgATP (syringe “II”) sufficient for a single turnover. The mixture is incubated to allow hydrolysis to occur (M-ATP → M-ADP-Pi) and then, in a subsequent mix, mixed with thin filaments (syringe “III”) containing either EGTA (Fig. 4, *B* and *C*) or added Ca(II) (Fig. 4, *D*–*G*). Conversion of acto-myosin-S1-ADP-Pi to acto-myosin-S1-ADP + Pi is tracked *via* a fluorescently labeled Pi binding protein (45). For these experiments native cardiac thin filaments are selected on account of their high ligand sensitivity (42). The authors point out that considerable homology exists between fast skeletal ELCA1 and cardiac ELCs (LC1a and b) in terms of chain length (>190 amino acids, 21, 000 g/mol) and sequence of the F-actin binding region (46, 47, 48).

Measurements featuring apo thin filaments (no bound ligands) will be described first. Owing to the high Ca(II) sensitivity of native cardiac thin filaments, the instrument syringes are washed with EGTA (in assay buffer, 5 mM Mops, 2 mM magnesium acetate, pH 7.00, T, 20 °C) beforehand. When this precaution is taken, the recorded change in fluorescence conforms to a slow monoexponential process for both types of myosin. Sample time traces are presented in Figure 5, *B* and *C*. The observed rates for a thin filament (actin monomer) flow cell concentration of 27 μM are between 0.66 to 0.8 s^−1^ (Fig. 5, *B* and *C*), indicating that the ligand-free thin filaments are “turned-off”. For certain batches of cardiac thin filaments, a small, rapid fluorescence increase is seen that contributes to ∼10% of the total signal. The problem could stem from a slight breakdown of troponin (Fig. 1 of (41)) that leads to a loss of regulation. A trace amount of endogenous Pi within the thin filaments is also possible even though purine nucleoside phosphorylase and 7-methylguanosine are added to all solutions beforehand.

When the measurements are repeated at pCa 4, the fluorescent signal follows a biexponential trajectory with time, consisting of a dominant fast component (∼80% of total amplitude) followed by one that is slower and smaller in magnitude (Fig. 5, *D* and *E*). The fast fluorescence change corresponds to Pi dissociation (*i.e.* regulated AM-ADP-Pi → regulated AM-ADP + Pi) (39, 42). At 27 μM thin filaments the rates of Pi dissociation are 55 s^−1^ (S1A1, Fig. 5*D*) and 40 s^−1^ (S1A2, Fig. 5*E*). But the difference between the two myosins narrows as the thin filament concentration is raised. For example, at 65 μM, values of approximately 70 s^-1^ are observed (Fig. 5, *F* and *G*), indicating that the rate *versus* concentration relationship for the A1-containing isoenzyme is near plateau whereas that of the A2 is not. The point is made clear in Figure 5*H*. Hyperbolic fits of the concentration dependences yield maximum extrapolated rates and *K*_*apps*_, respectively, of 81.4 s^−1^ and 8.9 μM for S1A1 (open symbols) and 129.3 s^−1^ and 57.6 μM for S1A2 (closed symbols) (Fig. 5*H*). At the highest concentrations of protein, it is apparent that Pi dissociation accounts for 90% of the total amplitude (Fig. 5*H*, upper panel). Note: a maximum rate of 27 s^−1^ was previously reported for cardiac myosin-S1 and native cardiac thin filaments (pCa 4, zero apomyosin-S1) (41). The difference between the current and previous measurements is explained by the kinetic properties of cardiac and skeletal myosins (49, 50, 51).

The dependence of the rate of Pi release on the concentration of turned-off thin filaments (pCa 8, zero apomyosin-S1) is shown by “x” symbols in Figure 5*H*. Titration is performed only with myosin-S1A2. Although the rates do not fit a clear hyperbola, the values at the three highest protein concentrations are: 0.66 s^−1^, 0.69 s^−1^, and 0.66 s^−1^—close to what has been reported for myosin-S1A1 (39, 40).

Summing up, Ca(II) binding to the cardiac thin filament instigates a pronounced acceleration in the maximum rate of Pi dissociation for both types of myosin-S1. At pCa 4, the longer the light chain, the tighter the interaction between myosin-S1 and thin filaments, the slower the maximum rate. Thus, it is evident that Pi dissociation is coupled to ELC binding.

### Effect of essential light chain isotype on the apomyosin-S1-accelerated rate of Pi dissociation from a native thin filament (pCa 8)-myosin-S1-ADP-Pi complex

**Figure 6:**
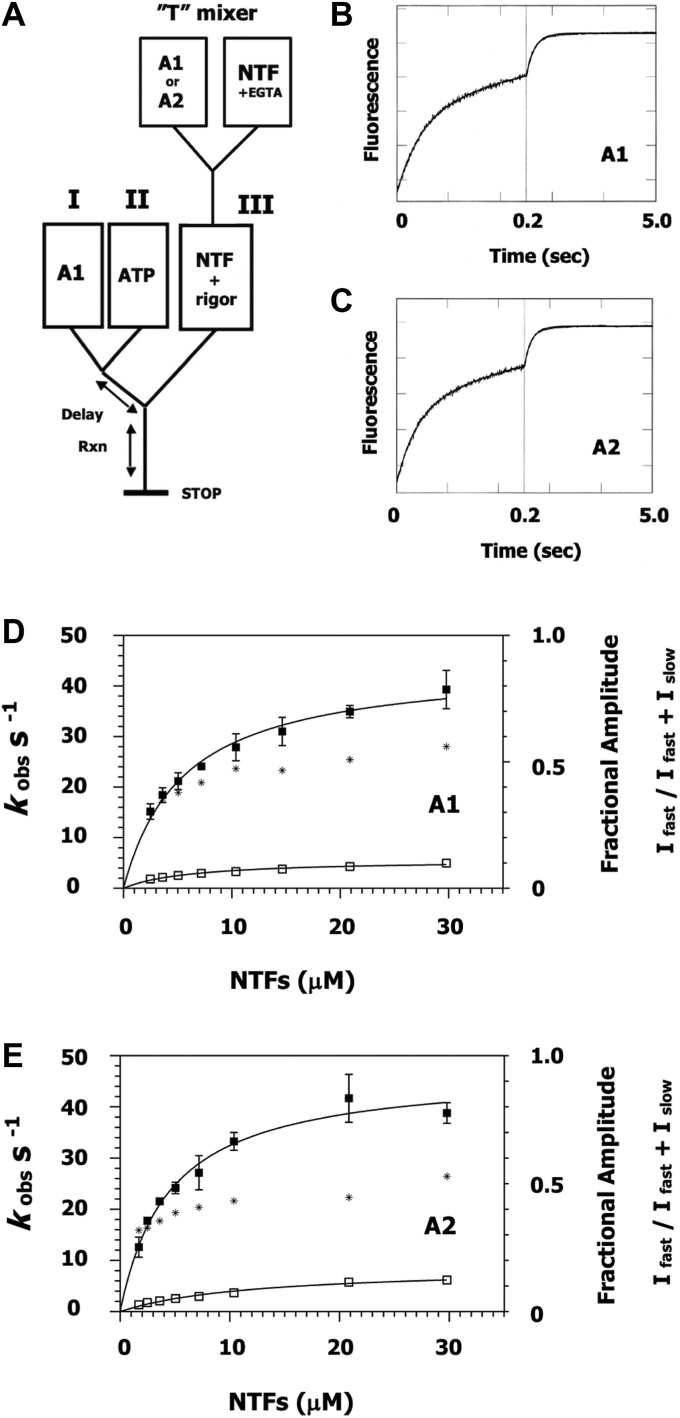
**Inorganic phosphate dissociation from a thin filament-myosin-S1-ADP-Pi complex in the presence of apomyosin-S1 and at pCa 8.***A*, instrument configuration same as in Figure 5*A* with the addition of a “T”-mixer for premixing native cardiac thin filaments (NTFs) and myosin-S1A1 or A2. Stoichiometry, 1 S1 per 7 actin monomers. The myosin-S1s are treated with PMSF prior to exposure to NTFs in order to kill any residual protease and protect the thin filaments. Comparison (nonblind) is performed on consecutive days. Syringe “I”, myosin-S1A1 (A1); syringe “II”, ATP and syringe “III”, premixed NTFs (pCa 8) + myosin-S1. Delay between mixes, 1 s. Buffer, 5 mM Mops, 2 mM Mg acetate, pH 7.00, 20 °C. *B* and *C*, time traces. As per Figure 5, Pi release is monitored over two different time windows each of 250 data points. Final concentrations in flow cell, 0.44 μM myosin-S1A1, 0.44 μM ATP, 10.3 μM NTFs, 1.1 mM EGTA and 5 μM phosphate binding protein + either myosin-S1A1 (*B*) or myosin-S1A2 (*C*). The presented traces are averages of recordings stemming from three or more shots (*i.e.* passes through the stopped-flow instrument) and are best fits to a given double exponential equation: *B*, I(t) = 0.49e^-26.4t^ + 0.51e^-3.3t^ + C. *C*, I(t) = 0.46e^-31.8t^ + 0.54e^-3.7t^ + C. *D* and *E*, concentration dependence. Three titrations are performed using two different batches of isomyosin-S1s and NTFs. Titration is performed systematically in descending order of concentration. Points are averages of at least nine shots. *t* test comparisons of the rates of Pi dissociation (A1 *versus* A2) indicate no significant difference: *p* > 0.1. *D*, apomyosin-S1A1. *Closed symbols*, fast increase in fluorescence (direct measure of the rate of Pi dissociation). *Open symbols*, slow increase in fluorescence. Interpretation of the slow phase is complicated by there being more than one possible route of breakdown of the M-ATP pool including attached hydrolysis (K _AH_, Fig. 1). Hyperbolic fitting provides the following parameters: *k*_fast_ = 44.5 s^−1^ and K_app_ = 5.6 μM; *k*_slow_ = 5.7 s^−1^ and *K*_*app*_ = 16.4 μM. Asterisk, the fractional amplitude of the fast phase, I _fast_/I _fast_ + I _slow_. *E*, apomyosin-S1A2. *Closed symbols*, fast increase in fluorescence. *Open symbols*, slow increase in fluorescence. Hyperbolic fitting provides the following parameters: *k*_fast_ = 47.1 s^−1^ and *K*_*app*_ = 4.5 μM; *k*_slow_ = 9.9 s^-1^ and *K*_*app*_ = 21.2 μM. *Asterisk*, the fractional amplitude of the fast phase, I _fast_/I _fast_ + I _slow_. NTF, native thin filaments.

The effect of apomyosin-S1 binding to the thin filament is investigated next. The experimental approach is illustrated by the instrument configuration in Figure 6*A*.

First, native thin filaments (pCa 8) are combined with a given type of myosin in the absence of ATP using a “T” mixer. A mole ratio of 1 S1: 7 actin monomers is selected in accordance with a previous study (39). These steps are taken to ensure a saturating amount of bound apomyosin-S1 ahead of the second mix. The resulting mixture is immediately loaded into syringe “III” for use in double-mixing stopped-flow as outlined above. Except in this instance, only myosin-S1A1 is used in syringe “I”, owing to its demonstrated high affinity for thin filaments (Fig. 5*H*). Again, all syringes are soaked in EGTA prior to the actual experiment.

Using the set up in Figure 6*A*, biexponential fluorescence increases are recorded over time (Fig. 6, *B* and *C*). At a native thin filament concentration of ∼10 μM, similar rates of Pi dissociation (represented by the faster phase) are observed: 26 s^−1^ (S1A1, Fig. 6*B*) and 32 s^−1^ (S1A2, Fig. 6*C*). The maximum extrapolated rates obtained from the concentration dependencies are virtually identical: 44.5 s^−1^ (S1A1, Fig. 6*D*) and 47^−1^ (S1A2, Fig. 6*E*), and significantly (*p* < 0.01) lower than that of Ca(II)-mediated activation (Fig. 5*H*). For both types of myosin, Pi dissociation accounts for approximately 50% of the total amplitude at the highest NTF concentration (Fig. 6, *D* and *E* asterisks), again in contrast to Figure 5*H* where ∼80% of the fluorescence change occurs rapidly. The difference may stem from a two-fold Ca(II)-induced change in K_AH_ (Fig. 1).

Summing up the findings in this section, ELC length does not significantly influence apomyosin-S1 activation of Pi dissociation at pCa 8. It is also evident that Pi dissociation is significantly less responsive to apomyosin-S1 binding to cardiac thin filaments than it is to Ca(II) binding.

## Discussion

Myosin in fast skeletal muscle contains two essential light chains (ELCA1 and ELCA2) that emanate from the MYL1 gene (25, 26). Owing to the stringent conditions (*e.g.* high pH) that were initially employed to incur their dissociation (52, 53), these subunits are also referred to as alkali “A” light chains. The first sequence determination (source, rabbit) reported a difference of just over 40 amino acids between A1 and A2 (9). It is now known that the length of the N-terminal extension in A1 (N-ELCA1) varies depending on the species (23). Structurally, the extension, which contains several prolines, is elongated (24). Positive charges within the first 10 amino acids are critical for interaction with F-actin (11, 12, 13, 14, 15, 16, 17, 19, 20, 21, 31, 32, 33, 34, 35, 36), likely to a different monomer than that of the heavy chain of the same myosin molecule (as illustrated in Fig. 5 of (22)). The shorter variant, A2, which lacks the binding site, is absent from other types of striated muscle (reviewed in (8)). Heart and slow skeletal muscle synthesize long versions of the ELC (>190 amino acids in human and rat, (46, 47)) that are homologous to fast skeletal A1 in terms of N-terminal sequence. For example, the first nine amino acids in mammalian atrial ELC and ventricle ELC: methyl_3_-A-P-K-K-P-E-P-K-K9 compare with those of skeletal ELCA1: methyl_3_-A-P-K-K-D-V-K-K-P9.

Herein, the functional relevance of ELC isomorphism is investigated in solution-based assays. Such an approach permits the observation of individual steps in a reaction pathway during a single turnover. In addition to the kinetic parameters presented in Table 1, the main points of the investigation are as follows: (i) myosin-S1A1 and S1A2 exhibit similar burst hydrolysis rates; (ii) the N-terminal extension in ELCA1 increases the probability of an actomyosin-limited hydrolysis mechanism, (iii) ELCA1 binding to thin filaments at pCa 4 and in the absence of bound apomyosin is coupled to Pi release, and (iv) Ca(II) is the main regulatory ligand in heart. These points are elaborated on in turn below.

(i) At a high concentration of myosin-S1, 100 μM, ATP binding (M + ATP → M-ATP) is not rate limiting and the chemical quench-flow method therefore affords a direct measure of the burst rate (M-ATP → M-ADP-Pi). Accordingly, the plots in Figure 3, *A* and *B* depict two well-separated phases with the faster phase corresponding to ATP cleavage. It is apparent that the rate of this step and the equilibrium constant of the hydrolysis reaction are similar for each isomyosin. For example, at saturating ATP, 400 μM (T, 20 °C), values of between 54 to 58 s^−1^ (burst rate) and 1.9 to 2.1 (K_eq_) are observed. It should be noted that ATP cleavage was not time-resolved in the original quench flow study of Taylor and Weeds 1977 (43). In agreement with the original study (43), we find that myosin-S1A1 and S1A2 hydrolyze MgATP at essentially the same steady state rate, 0.027 s^−1^-0.029 s^−1^.
(ii) First demonstrated in 1975 (10), the two fast skeletal muscle myosins have been widely reported to be differentially activated by actin. The difference in activation is apparent below 50 mM ionic strength (54, 55). Under the conditions used herein, ∼10 mM ionic strength and 20 °C, the steady state rate of ATP hydrolysis for myosin-S1A1 peaks at low concentrations of unregulated F-actin (Fig. 4*A*) and also NTFs at pCa 4 (Fig. 4*B*). Then, upon further concentration increase, the rate declines in both instances (Fig. 4, *A* and *B*, closed symbols). By comparison, the relationship for myosin-S1A2 is hyperbolic over the same concentration range (Fig. 4, *A* and *B*). The projected A2 maximum rates, 7.6 s^−1^ and 10.1 s^−1^, exceed those of the A1-containing isoenzyme but a higher concentration of actin is needed for the maximum to be attained (Table 1). The observations are explained firstly by the N-terminal extension increasing the affinity of myosin-ATP for F-actin and secondly by the maximum steady state hydrolysis rate not being limited by a single kinetic step but by actin having contrasting effects on the rates of ATP bond scission (AM-ATP → AM-ADP-Pi) and Pi dissociation (AM-ADP-Pi → AM-ADP + Pi) (44). Thus, increasing the actin monomer concentration as per Figure 4, *A* and *B* instigates a transition from a myosin-limited ATP hydrolysis mechanism (faster cleavage *k*_H_, slower Pi dissociation *k*_-DP,_ lower pathway in Fig. 1) to one that is actomyosin-limited (slower bound cleavage *k*_AH_, faster Pi dissociation *k*_-DAP_, upper pathway Fig. 1). It follows that at low ionic strength, the N-terminal extension will cause a higher fraction of A1 heads to traverse the attached pathway as compared to A2 heads, leading to the observed decline in steady-state ATPase (Fig. 4, *A* and *B*). A bimodal concentration-dependence can be envisioned for A2 but solution experiments are limited by protein concentration, which is not the case physiologically.

**Table 1:** Summary of kinetic parameters

Process	S1A1	S1A2
MgATPase	2.8 s^−1^ a (SD 0.2)	7.6 s^−1^ b (SD 1.0)
F-actin	1.8 μM (SD 0.2)	6.4 μM (SD 0.5)
	2.3 s^−1^ b (SD 0.4)	
	12 μM (SD 5.0)	
MgATPase	5.2 s^−1^ a (SD 1.0)	10.1 s^−1^ b (SD 1.5)
NTFs pCa 4	1.0 μM (SD 0.3)	5.5 μM (SD 1.0)
	4.0 s^−1^ b (SD 1.0)	
*k*_-DAP_	81.4 s^−1^ (SD 3.9)	129.3 s^−1^ (SD 10.4)
pCa 4	8.9 μM (SD 1.7)	57.6 μM (SD 8.7)
*k*_-DAP_	44.5 s^−1^ (SD 2.2)	47.1 s^−1^ (SD 2.6)
pCa 8 + rigor		
*k*_H_	58 s^−1^	54 s^−1^

Tabulated values of maximum rate and concentration at half maximum rate. *a*, highest steady state rates for A1-containing myosin-S1 at low actin monomer concentration (Fig. 4, *A* and *B*, closed symbols, <10 μM). *b*, extrapolated steady-state MgATPase rates at high actin monomer concentration (Fig. 4, *A* and *B*). Pi dissociation, *k*_-DAP_ (Fig. 1), obtained from the fast fluorescence change observed in double-mixing stopped-flow (Figs. 5 and 6). Standard deviations, SD, are provided in parentheses. ATP cleavage, *k*_H_ (Fig. 1), obtained from quench-flow at 100 μM myosin-S1 + 25 μM ATP (Fig. 3, *A* and *B*, red triangles). Rigor signifies apomyosin-S1.

Three major dimeric myosins exist in fast skeletal muscle (5, 6, 7). In the case of A1A1 myosin, the expectation is that the additional actin binding residues increase the likelihood of the ATP cleavage step occurring while the head is filament bound, thereby supporting the maintenance of force (due to increased cross-bridge life time). By the same token, the weaker binding A2A2 myosin can be expected to conform to the main Lymn-Taylor pathway (56) where the hydrolysis step occurs mainly in the detached state. In this instance, the A2 isoform would support a faster contraction velocity (due to a faster cycle time). In the case of A1A2 myosin, it is reasonable to speculate that the A1-containing head will give the muscle fiber contractile properties more like the A1 homodimer than the A2 homodimer. All isoform combinations will on average detach and reattach many times during the hydrolysis of each ATP because myosin-ATP and myosin-ADP-Pi bind to, and dissociate from, thin filaments more rapidly than the hydrolysis and product dissociation steps.

(iii) Sequential (double-mixing) stopped-flow fluorescence (Figs. 5*A* and 6*A*) in combination with a Pi indicator protein allows aging of a pre-power stroke complex (AM-ADP-Pi → AM-ADP + Pi) to be monitored in real time. Previous application of this technique, in effect an *in vitro* copy of a working stroke, has demonstrated that the association of ligands (Ca(II) and apomyosin) to thin filaments causes a marked acceleration in the rate of Pi dissociation (reviewed in (42)).

Again, owing to the salt sensitivity of myosin binding to actin, it is necessary to perform such measurements in a solution of low ionic strength. For example, at near-physiological ionic strength, the rate of Pi dissociation is linearly dependent on the thin filament concentration and the maximum rate cannot be determined (57). In the current crop of double-mixing experiments (with acetate as Mg(II) counterion), at low Ca(II) ELC length did not significantly contribute to the degree of activation of Pi dissociation by apomyosin-S1 (Fig. 6, *D* and *E* and Table 1). But it does at pCa 4 in the absence of apomyosin (Fig. 5*H*). For this ligand condition the measured parameters are the following: 81.4 s^−1^ (S1A1, K_app_ 8.9 μM) *versus* 129.3 s^−1^ (S1A2, K_app_ 57.6 μM) (Table 1). A maximum rate of ∼80 s^−1^ is comparable to the value obtained with S1A1 and unregulated actin (39), and illustrates the pronounced Ca(II) sensitivity of the cardiac regulatory system. The fact that S1A2 exceeds this rate indicates that ELCA1 docking to thin filaments (pCa 4) is coupled to Pi release (or a preceding conformational change, (58)). On this point, coupling may involve, in part, association of the heavy chain’s SH3 domain with proline residues in A1 (59). It should be noted that the six to seven-fold difference in filament concentrations at half maximal rate may be an underestimate of the disparity in the actual K_ds_ (60).

How do points (ii) and (iii) line up with the results of other studies? Although ELCA1 affinity for F-actin is considered weak (54, 55), there is ample evidence to suggest that it persists in muscle where the sarcomeric proteins exist at exceedingly high concentration and are arranged in a lattice. Evidence includes the following: the increase in skinned fiber shortening velocity (34) and myofibrillar ATPase activity (35) by synthetic N-ELCA1 peptides; the dependency of unloaded shortening velocity of skinned fast skeletal muscle fibers on light chain composition, specifically fibers containing a preponderance of ELCA1 (*i.e.* high A1/A2 ratio) display comparatively slower shortening (61, 62); the lowering of force in transgenic heart bundles by residues 5 to 14 of ELCA1 (63) (which again presupposes the competitive displacement, and therefore presence, of filament bound holo ELCA1) and the correlation of age-related decline in ELCA2 content (and therefore an age-related enhanced influence of ELCA1) and muscle slowing (64). Further, the observed isoform-related differences in the rates of steady-state ATP hydrolysis (Fig. 4, *A* and *B* and Table 1) and Pi dissociation (Fig. 5*H* and Table 1) are compatible with the reported faster movement of F-actin by myosin-S1A2 compared to S1A1 in a motility assay (27).

(iv) The results of double-mixing experiments show a significant difference between the Ca(II)-mediated and apomyosin-mediated maximum rates of Pi dissociation; nearly two-fold for S1A1 (81.4 s^−1^ *versus* 44.5 s^−1^, p < 0.01) and more than two-fold for S1A2 (129.3 s^−1^ *versus* 47.1 s^−1^). These results point to a system of regulation in heart that primarily involves the association and dissociation of calcium to and from the thin filament. That is, any rigor heads that materialize during contraction will provide only an incremental acceleration of the rate of Pi release (42). The authors do not think that their observations are explained by an insufficient number of nucleotide-free heads, as a ratio of one to seven actins is used in the premix (Fig. 6*A*). Previously (Fig. 5 of (39)) a stoichiometry of 1 myosin-S1A1 per 14 actin monomers was found to be sufficient for maximal rigor activation of reconstituted skeletal muscle thin filaments (pCa 8), preparations that are less responsive to the binding of ligand than native cardiac thin filaments. Premixing (Fig. 6*A*) is presumed to yield a random distribution of rigor heads. While it is not possible to exclude a subsequent reorganization of heads, one that would lead to less activation due to regulatory units being empty or congested, the current results compare to those of previous experiments involving cardiac thin filaments (41, 42) where a surplus of apomyosin-S1A1 over ATP was used in the first mix. From this, we conclude that if a redistribution of heads had taken place, it had little bearing on the measurements.

Ca(II) as main regulatory ligand is consistent with the report of Sun *et al* where at low pCa the contractility of cardiac fibers did not require apomyosin (65). Further, in a normal beating heart most of the myosin molecules reside in a folded conformation (66, 67, 68) and the number of cycling heads is small (67). But it does not align with predictions of the three-state model (69) which was formulated from experiments where ATP is absent. On this point, the authors had previously shown that regulation depends on myosin’s active site nucleotide (41, 42), which ties with the nucleotide-sensitive conformation of the critical connector segment switch II (reviewed in (70)). Specifically, whereas myosin-ADP-Pi is the power stroke generating intermediate (closed switch II), nucleotides such as ADP that stabilize the post power stroke conformation (open switch II) do not produce a power stroke on binding to actin (71).

The high Ca(II) sensitivity of the cardiac regulatory system demonstrated herein can be considered real. While mammalian cardiac and skeletal actins share only four substitutions (72, 73), troponin-I and the major variant of troponin-T in heart are both longer than their fast skeletal counterparts (73, 74, 75). When tropomyosin is largely unphosphorylated, the N-terminal domain of troponin-T (N-Tn-T) stabilizes a turned-off thin filament state (first reported in (76)). Positionally, N-Tn-T stretches from the troponin core and spans the overlap region of tropomyosin (77, 78, 79, 80). Then, each N domain of the cardiac isoform attaches to the opposite tropomyosin strand thereby forming a cross-brace (81). The resulting molecular scaffold conceivably underpins the Ca(II) response demonstrated herein (Fig. 5) and elsewhere (82, 83, 84).

In conclusion, the regulatory properties of the cardiac thin filament, which include one low affinity site in troponin-C (85), align with the high frequency control of myosin in the heart and cater for beat-to-beat contractility. The N-terminal extension in ELCA1 increases myosin head affinity for actin, enforcing comparatively more flux through the slower actin “inhibited” arm (44) of the hydrolysis pathway and reduces the maximum rate of decomposition of the pre-power stroke complex (Fig. 1). The predicted outcomes are comparatively slower contraction and higher force. Lacking the extension, ELCA2 supports faster contraction and less force by favoring flux through the faster (detached) hydrolysis arm and promoting a faster maximum rate of Pi dissociation. These findings, which align with the light chain content of different striated muscles (8), provide a biochemical understanding of how the N-terminal extension in essential light chain A1 influences muscle performance, a theme that began half a century ago (3).

## Experimental procedures

### Proteins

Typically, ∼10g of myosin (source, 1 kg of bulk rabbit skeletal muscle), is digested with chymotrypsin (equivalent of 200 mg of myosin to 1 mg of protease). The reaction (8 min, 20 °C) is terminated with lima bean trypsin inhibitor (2 mg per mg of chymotrypsin). Digestion produces a myosin-head fragment that retains a full length essential light chain whereas the regulatory light chain is degraded (10). The yield is ∼20%. Undigested myosin is removed by centrifugation. Myosin-S1 is separated into the ELCA1 and ELCA2 containing fractions by diethylaminoethyl-52 cellulose column chromatography (10). Fractions are screened by electrophoresis (86), pooled, and the protein collected by precipitation in 50% (mass/vol) ammonium sulfate. Then, the pellets are dispersed in a minimal volume of buffer containing 5 mM Mops, 2 mM MgCl_2_, 1 to 2 mM DTT set to pH 7.00 at 4 °C and dialyzed against several changes of the same buffer in the cold. Following clarification by centrifugation at 50,000 rpm for 15 min in a Beckman 70 Ti fixed-angle rotor, the supernatants are drip frozen in liquid nitrogen and stored at minus 80 °C. Native thin filaments are prepared from pig ventricle as described in reference (87) and dialyzed against several changes of assay buffer containing 1 to 2 mM DTT in a cold room. Actin is isolated from bulk rabbit skeletal muscle by polymerization-depolymerization (88). Concentrations are obtained spectrophotometrically after correction for light scattering, 1.5 × A_320nm_ using the following A_280, 1mg/ml_ extinction coefficients, myosin-S1, 0.79 ml/mg and native cardiac thin filaments, 0.7645. In the latter case, an estimate of 1/7th of the molar mass of a regulatory unit, 63,000 is used to convert to the actin subunit concentration. Therefore, a corrected *A*_280nm_ of 7.2 is equivalent to a [NTF] of ∼150 μM.

Chemicals are purchased from Sigma-Aldrich.

### Steady state MgATPase

The extent of hydrolysis is obtained by spectroscopic measurement of Pi (89). Buffer conditions, 5 mM Mops, 2 mM MgCl_2_, 1 mM MgATP, pH 7.00, 20 °C. Ionic strength, 10.5 mM. Reaction is initiated by the addition of myosin-S1.

### Double-mixing stopped-flow

Inorganic phosphate transients are tracked using a coumarin-labeled Pi binding protein (44, 45) and a KinTek model SF-2001 double-mixing stopped-flow fluorimeter (39). Instrument configurations are shown in Figures 5*A* and 6*A*. Excitation at 430 nm and emission >450 nm. Buffer conditions, 5 mM Mops, 2 mM Mg acetate, pH 7.00, 20 °C.

Ca(II)-mediated activation of Pi dissociation is investigated by first combining a given myosin-S1 isoenzyme (syringe “I”) and ATP (syringe “II”). The resulting steady state mixture is held in a delay line for 1 s and then, in a second mix, combined with native thin filaments containing either 0.5 mM CaCl_2_ for pCa 4 or 2 mM EGTA for pCa 8 (syringe “III”) (Fig. 5*A*). The dilution of the syringe contents is 2:9 for myosin-S1 (volume of syringe “I” = 2 ml) and ATP (volume of syringe “II” = 2 ml) and 5:9 for thin filaments (volume of syringe “III” = 5 ml). All syringes are soaked with assay buffer containing a Pi scavenging system consisting of purine nucleoside phosphorylase and 7-methylguanosine (39). The scavenging system is also added to native thin filaments ∼1 h prior to use.

Apomyosin-S1-mediated activation of Pi dissociation is investigated by premixing native thin filaments (pCa 8) with a given myosin-S1 isoenzyme, pretreated with PMSF in order to protect regulatory proteins from any remaining chymotrypsin in a 1/7 M ratio relative to actin monomer using a “T” mixer. The resulting apoS1-thin filament mixture is then immediately inserted into syringe “III”. The experiment is performed with myosin-S1A1 in syringe “I” (Fig. 6*A*).

A minimum of three shots through the stopped-flow are performed. Two time windows each of 250 data points are selected for the appropriate lengths of time, as indicated in the Figures. Micromath Scientist graphing software provided with the KinTek SF-2001 instrument is used to fit the averaged data traces to one or two exponential terms and obtain rate constants and standard deviations. Additional details of experiments involving Pi binding protein are available in 40.

### Statistical analysis

Significance is assessed by independent two sample *t* test.

### Quench-flow

Chemical quench measurement of the hydrolysis of ATP is carried out using a custom-built apparatus. Buffer conditions, 50 mM potassium acetate, 10 mM Mops, 3 mM MgCl_2_, pH 7.00, 20 °C. Equal volumes of myosin-S1A1 or S1A2 (containing 1–2 mM DTT) and ATP (containing ∼2 × 10^4^ dpm of [^32^P]-gamma ATP) are mixed, held in a delay line for the desired time and then quenched with 2 M HCl to give a final volume of 1.0 ml. The total radioactivity of ATP in each sample is determined by counting 0.3 ml directly. Unhydrolyzed ATP is removed in the following manner: a 0.6 ml portion of the sample is mixed with an equal volume of a 10% (mass/vol) charcoal slurry in 2 M HCl + 0.35 M sodium phosphate, chilled on ice and centrifuged. Fractional hydrolysis is obtained from the ratio of the charcoal-treated and total counts after background subtraction.

## Data availability

All data are contained within the text of the article.

## Conflict of interest

The authors declare that they have no competing interests**.**
